# Symptomatic Maxillary Sinus Retention Cyst Following a Prior Sinus Perforation: A Case Report

**DOI:** 10.1155/crid/1849358

**Published:** 2025-06-06

**Authors:** Arta Sinanaj Demiri, Shaqir Demiri, Edin Demiri, Besir Salihu

**Affiliations:** ^1^Department of Oral Surgery, Private Polyclinic “Aesthetica”, Mitrovica, Kosovo; ^2^Department of Otorhinolaryngology, Private Polyclinic “Aesthetica”, Mitrovica, Kosovo; ^3^Department of Oral Surgery, University Clinical Center of Kosovo, Pristina, Kosovo

**Keywords:** Caldwell–Luc, cholesterol crystals, chronic sinusitis, odontogenic complications, retention cyst, sinus surgery

## Abstract

Maxillary sinus retention cysts (MSRCs) are benign, fluid-filled lesions most often discovered incidentally on radiographic imaging. Although typically asymptomatic, larger cysts may lead to clinical symptoms due to pressure on surrounding sinus structures. This report presents a rare symptomatic case of an MSRC in a 70-year-old female patient with a prior history of sinus perforation following upper molar extraction. The patient experienced severe unilateral facial pain, nasal congestion, debilitating headaches, and dizziness over a 6-month period. Radiographic imaging (CT scan) revealed a well-circumscribed, dome-shaped lesion in the right maxillary sinus without signs of bone erosion. Given the failure of conservative management and the intensity of symptoms, surgical intervention via the Caldwell–Luc approach was performed. Intraoperatively, the cystic lesion was enucleated and sent for histopathological examination. Histology confirmed a benign retention cyst lined with respiratory epithelium, along with inflammatory infiltrates and the unusual presence of cholesterol crystals, which is an uncommon finding in MSRCs. The patient's history of sinus perforation likely contributed to chronic inflammation and cyst formation. This case emphasizes the importance of considering dental history, particularly previous maxillary molar extractions with sinus involvement, in patients presenting with atypical sinus symptoms. While endoscopic sinus surgery is often the preferred approach, the Caldwell–Luc procedure remains valuable in selected cases with complex anatomy or previous surgical alterations. Clinicians should be aware of rare presentations of MSRCs that may require tailored surgical management for symptom resolution and recurrence prevention.

## 1. Introduction

Cystic lesions of the orofacial region encompass a wide variety of entities, both odontogenic and nonodontogenic in origin [1]. Odontogenic cysts, characterized by fluid accumulation within a distinct cavity, represent some of the most commonly encountered lesions affecting the oral and maxillofacial structures [2]. Their development is primarily driven by the activation and multiplication of residual odontogenic epithelial cells embedded in the jawbones, particularly remnants like the epithelial rests of Malassez and Serres [3]. Of the odontogenic cysts, the most common are radicular, dentigerous, and odontogenic keratocysts [4].

Nonodontogenic cysts such as nasopalatine duct cysts, nasolabial cysts, and retention cysts such as mucoceles are also present in the orofacial region [1]. The nasopalatine duct cyst is the most common nonodontogenic cyst of the jaw, typically presenting as a radiolucent lesion in the midline of the anterior maxilla. These are usually managed via surgical enucleation and rarely recur [5]. Mucoceles, although more common in the minor salivary glands, can also affect the paranasal sinuses when they become obstructed and expand, potentially leading to bone remodeling or erosion [6].

Similar to mucoceles, maxillary sinus retention cysts are benign lesions, filled with fluid, commonly found in the maxillary sinuses, often identified spontaneously during imaging procedures [7]. These cysts develop when the ducts of mucous glands within the sinus lining become obstructed, which leads to mucus accumulation and cyst formation [8]. Their prevalence in the general population ranges from 3% to 35% [9–11], depending on the study and the population examined. In our experience, most of the cysts are usually asymptomatic; however, larger cysts may cause nasal obstruction, facial pain, or sinus pressure, especially if they fill the sinus space and exert pressure on the mucosal lining. If they are located in the ostium, they may obstruct the sinus opening, potentially leading to infection [10]. In some cases, this pressure can cause headaches and periorbital pain.

The precise cause of maxillary sinus retention cysts is not fully understood, but the primary mechanism is suspected to be the blockage of seromucous glands within the Schneiderian membrane. Factors that contribute to this blockage include chronic sinus inflammation, allergic reactions, trauma, and local irritants [12]. Odontogenic causes, which usually originate from dental or periodontal disease, have also been explored due to the close anatomical relationship between the maxillary teeth and the sinus floor [13]. A careful preliminary differential diagnosis should be made between maxillary sinus retention cysts and odontogenic sinusitis, which represents 25% to 40% of maxillary sinusitis [14].

As for the possible odontogenic correlations, the roots of the upper posterior teeth, particularly the molars and premolars, are often in very close proximity to, or penetrate, the maxillary sinus floor. Pathologies such as periapical infections, periodontal disease, or complications from dental procedures such as traumatic extractions, implants, or root canal therapy can potentially affect the sinus [7, 14]. Odontogenic infections, in particular, can incite localized inflammation and interfere with the function of the mucous glands, thereby facilitating the formation of mucous retention cysts. Differentiating these kinds of cysts from other sinus or odontogenic pathologies, including mucoceles and dentigerous cysts, is essential to avoid unnecessary treatment. Radiographically, mucous retention cysts appear as dome-shaped, noncorticated opacities and are often asymptomatic [15–17], whereas dentigerous cysts typically present as well-defined, corticated radiolucencies enveloping the crown of an impacted tooth. Case reports have described instances where ectopic maxillary third molars became embedded within the sinus and developed into dentigerous cysts, underscoring the importance of recognizing their odontogenic origin and distinguishing them from nonodontogenic mucosal lesions [18, 19].

Histopathologically, MSRCs are composed of a cystic cavity lined by pseudostratified ciliated columnar epithelium, typical of respiratory mucosa. The cysts contain serous or mucous fluid and do not exhibit signs of inflammation or infection unless secondarily involved. The epithelial lining stays intact, and the adjacent maxillary sinus mucosa may show mild inflammation if present [20]. These cysts are mostly nonexpansile and do not affect the surrounding bone structure or cause displacement of the sinus walls [21].

This case report highlights a unique instance of a maxillary sinus retention cyst containing cholesterol crystals, discovered in a patient with a positive history of sinus perforation during dental procedures. The findings underscore the importance of recognizing atypical presentations and their potential link to prior surgical interventions, contributing to our understanding of cyst formation and management in the context of dental care.

## 2. Case Presentation

A 70-year-old retired female teacher presents in our clinic with severe, unrelenting facial pain and debilitating headaches that have progressively worsened over the past 6 months. She described the pain as an intense crushing pressure localized to the right side of her face, radiating into her head and upper jaw, leaving her unable to perform even basic daily activities.

She also declared that each time she bent forward, the pressure surged, as if something was trapped inside her sinuses, worsening her discomfort. The pain was so intense that it disrupted her sleep and left her exhausted during the day. Along with the pain, she has suffered from constant nasal congestion and postnasal drip. Her most troubling symptom, however, was constant episodes of dizziness that left her disoriented and anxious.

The patient reported a significant history of dental complications; notably, she had undergone the extraction of her upper right first molar 10 years ago in order to receive complete dental prosthesis treatment, which resulted in a sinus perforation.

This complication, according to the patient, had been managed at the time but lingered in her memory as the possible cause of her persistent and worsening symptoms.

On physical examination, the patient appeared in distress, visibly uncomfortable due to the constant pressure in her face. Her vital signs were stable, with a blood pressure of 130/85 mmHg, a heart rate of 76 bpm, and a respiratory rate of 16 breaths per minute. She was afebrile.

On endoscopic examination, there was marked tenderness over the right maxillary sinus. The nasal mucosa appeared congested, though there were no signs of purulent discharge or polyps. On examination of her oral cavity, the patient was completely edentulous and her past history of sinus perforation was barely noted clinically.

On palpation, the portion of the palate corresponding to the sinus and lesion was highly sensitive, while the vestibular area showed reduced sensitivity. However, percussion of the maxillary sinus elicited unbearable sensations, indicating significant discomfort in that region.

The oropharynx was clear, and her eye examination showed no abnormalities, despite the intensity of her facial pain.

Given the severity of her symptoms, the patient underwent a radiographic examination.

**Figure 1:**
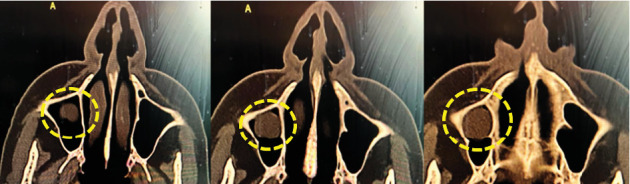
CT scan showing a well-defined, dome-shaped retention cyst in the right maxillary sinus. The cyst is exerting light pressure against the sinus walls, likely correlating with the patient's reported symptoms of extreme discomfort.

A subsequent CT scan confirmed the presence of a well-defined, dome-shaped retention cyst in the right maxillary sinus (Figure 1). The cyst was pressing against the sinus walls, likely contributing to the extreme pressure she was feeling. Importantly, no signs of bone erosion or involvement of adjacent structures were seen, differentiating it from more invasive or malignant processes.

After the initial diagnosis, the patient underwent surgical intervention. Given her history of sinus perforation following the extraction of the upper right first molar, and the dramatic clinical presentation, a Caldwell–Luc procedure was performed as the most appropriate approach.

**Figure 2:**
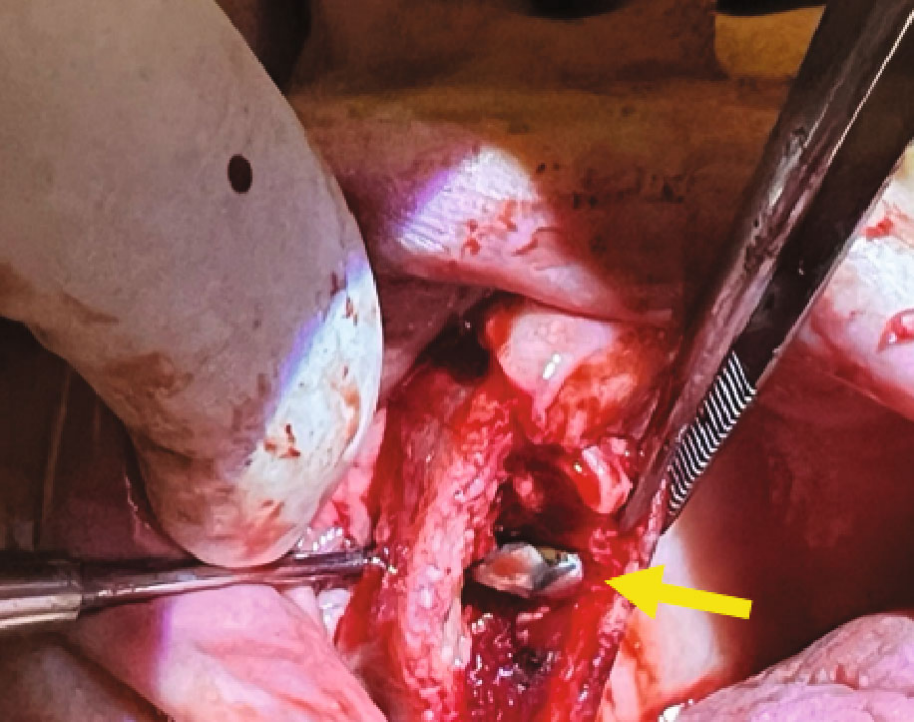
Intraoperative view following the surgical removal of the cyst through a lateral window using the Caldwell–Luc procedure, revealing the prior communication between the sinus and the oral cavity.

During the surgery, a bony window was created in the anterior wall of the maxillary sinus, allowing for full access to the affected area. The cystic lesion was completely enucleated, ensuring the sinus cavity was thoroughly cleared (Figure 2).

**Figure 3:**
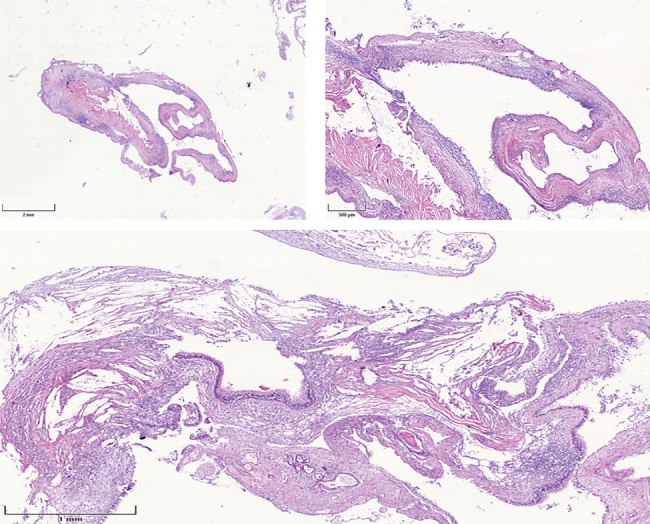
In the examined tissue samples, the luminal surface of the cystic formation is identified, demarcated by cylindrical epithelium, either mucinous or pseudostratified, exhibiting reactive and erosive changes with atypia. Edematous alterations and areas of mixed inflammatory infiltrates are observed in the stroma. At the periphery, several islands of calcified osteoid are identified. There are focal areas of degenerative changes and cholesterol crystals.

After the cystic lesion was completely enucleated, the excised tissue with a volume of 2.3 × 1.5 × 0.8 cm was sent for histological examination. The microscopic analysis confirmed the diagnosis of a benign maxillary sinus retention cyst, characterized by a cystic lining of respiratory epithelium with no signs of dysplasia or malignancy (Figure 3). The analysis confirmed the presence of inflammatory cells and cholesterol crystals surrounded by giant cells and macrophages.

## 3. Discussion

In our case study, we briefly described both the clinical and histological characteristics of a patient with a maxillary sinus retention cyst. While in the literature it was reported that most cases are asymptomatic and typically do not require treatment, our case presented with severe and dramatic symptoms that necessitated intervention.

Various authors have documented the clinical signs associated with MSRCs, highlighting the correlation between these cysts and symptoms such as headaches, nasal obstruction, facial pain in the sinus regions, postnasal drip, and nasal discharge [22–24]. Wang et al. found nasal obstruction in 52.5%, nasal discharge in 35.7%, and headaches in 2.5% of their MSRCs [22], while in Hadar et al.'s study on symptomatic retentive cysts, headaches were reported in 63% of patients [23]. Similarly, Busaba and Kieff reported that all of their patients with MSRCs experienced facial pain or pressure in the sinus area [24].

The radiographic features of the patient's cyst were similar with those described in other studies [25, 26]. However, it was the histological findings that set this case apart from the typical presentation of MSRCs.

Cholesterol crystals are not commonly associated with maxillary sinus retention cysts, but they can occasionally be found in long-standing or chronic cystic lesions of odontogenic origin [27]. These crystals form as a result of the breakdown of lipids and red blood cells in areas of chronic inflammation or fluid stasis. Their presence is more commonly seen in conditions such as cholesterol granulomas [28–30], but in retention cysts, they are rare because these cysts typically do not undergo the level of chronic inflammatory changes needed to generate the breakdown products that lead to crystal formation. Therefore, our study findings support the notion that prolonged inflammation, possibly due to previous surgical interventions or infections, can lead to the formation of cholesterol crystals within sinus cysts.

The relationship between maxillary sinus cysts and previous dental procedures, particularly sinus perforation following tooth extraction, is well documented. Sinus perforation can disrupt normal sinus drainage, leading to mucous retention and the potential formation of cystic lesions [7]. In our case, the initial diagnosis was challenging due to a preliminary suspicion of a postoperative maxillary cyst (POMC), also known as a surgical ciliated cyst. These lesions often develop following radical sinus surgery, orthognathic surgery, sinus elevation, or midfacial fracture repair [31]. Additionally, we had to consider and exclude the possibility of a postoperative maxillary mucocele (POMM), which, as reported by Ku et al., is a delayed complication typically arising after radical maxillary sinus surgery, particularly the Caldwell–Luc procedure [32]. In our patient, the past dental history likely played a role in the development of a retention cyst. Her symptoms were further aggravated by chronic inflammation and cyst enlargement, consistent with findings by Siwach et al. in a similar case involving a POMC [33].

In most cases, MSRCs are asymptomatic and treatment is not required. However, regular follow-up with imaging is recommended to monitor any changes in size or symptoms [7].

If the cyst is odontogenic in origin or associated with dental pathologies, treatment of the underlying dental condition is typically warranted. In rare cases where MSRCs become symptomatic or significantly enlarge, surgical intervention, such as endoscopic sinus surgery, or more invasive procedures such as the Caldwell–Luc surgical technique may be considered [32].

The decision to perform the Caldwell–Luc procedure was based on the patient's clinical history and the failure of conservative measures to alleviate her symptoms. While less invasive endoscopic techniques are often favored in modern practice, the Caldwell–Luc procedure provided the necessary access to completely enucleate the cyst, especially given her history of sinus perforation. This approach allowed for thorough removal of the cyst and ensured proper drainage of the maxillary sinus, preventing recurrence.

A more detailed information outlining various treatment approaches for maxillary sinus retention cysts, along with their indications, advantages, and disadvantages is presented in Table 1.

## 4. Conclusion

This case highlights the interesting, unusual, and almost atypical symptomatic presentation of a maxillary sinus retention cyst in a patient with a history of sinus perforation after dental extraction.

The identification of cholesterol crystals in the cyst underscores the chronic inflammatory changes that may occur in such lesions. While most maxillary sinus retention cysts remain asymptomatic and do not require treatment, surgical intervention, such as the Caldwell–Luc procedure, may be necessary in cases with severe symptoms and prior history of sinus perforation of odontogenic origin.

This case emphasizes the need for careful evaluation of dental histories and the use of appropriate surgical techniques to ensure optimal patient outcomes.

## Figures and Tables

**Table 1:** Treatment approaches for maxillary sinus retention cysts (MSRCs).

**Treatment approach**	**Indications**	**Advantages**	**Disadvantages**
Observation	Asymptomatic cysts	Non-invasive, avoids surgical risk	Requires regular follow-up, potential for cyst growth and complications
Functional endoscopic sinus surgery	Symptomatic cysts associated with chronic sinusitis, larger cysts that cause nasal obstruction	Minimally invasive, allows direct visualization and access to the sinus, may treat multiple sinus issues simultaneously	Risks of bleeding, infection, or damage to surrounding structures, requires general or local anesthesia
Caldwell–Luc procedure	Large symptomatic cysts, failed conservative treatment, coexisting odontogenic infection	Direct access to the cyst, can remove the cyst and address dental pathology simultaneously	Very invasive, risk of complications (oroantral fistula, nerve damage), longer recovery time
Dental treatment (root canal therapy or extractions)	When the cyst is odontogenic in origin or accompanied by dental infections	Addresses underlying cause while alleviates the symptoms if dental pathology is treated	May not resolve cyst if it is not directly related to the dental issue, potential for dental complications
Steroid injections	Inflammation associated with cysts, adjunctive treatment for symptoms	Minimally invasive, can reduce inflammation and improve symptoms	Temporary relief, does not remove the cyst, potential side effects from steroids

## Data Availability

The data that support the findings of this study are available on request from the corresponding author. The data are not publicly available due to privacy or ethical restrictions.
